# Syntool: A Novel Region-Based Intolerance Score to Single Nucleotide Substitution for Synonymous Mutations Predictions Based on 123,136 Individuals

**DOI:** 10.1155/2017/5096208

**Published:** 2017-07-24

**Authors:** Tongda Zhang, Yiran Wu, Zhangzhang Lan, Quan Shi, Ying Yang, Jian Guo

**Affiliations:** ^1^BGI Education Center, University of Chinese Academy of Sciences, Shenzhen, Guangdong 518000, China; ^2^BGI-Shenzhen, Shenzhen, Guangdong 518083, China; ^3^China National Genebank-Shenzhen, BGI-Shenzhen, Shenzhen, Guangdong 518083, China; ^4^Hong Kong Bioinformatics Centre, The Chinese University of Hong Kong, Shatin, Hong Kong; ^5^BGI-Tech, BGI-Shenzhen, Shenzhen 518083, China

## Abstract

**Background:**

Synonymous mutation is the single nucleotide change that does not cause an amino acid change but can affect the rate and efficiency of translation. So recent increase in our knowledge has revealed a substantial contribution of synonymous mutations to human disease risk and other complex traits. Nevertheless, there are still rarely synonymous mutation prediction methods.

**Methods:**

Nonsynonymous and synonymous coding SNPs show similar likelihood and effect size of human disease association. Here we defined synonymous and missense variation as single nucleotide substitution variation. And then we evaluated the intolerance of genic transcripts to single nucleotide substitution variation based on gnomAD 123136 individuals. After regressing all variations on common variations, we defined residuals of regression model as every genomics region intolerance scores.

**Results:**

We constructed a total of 24799 nonoverlapped region-based intolerance score by their intolerance to single nucleotide substitution variation (Syntool). The results show that Syntool score can discriminate synonymous disease causing mutations in Human Gene Mutation Database (HGMD Professional) and ClinVar database much better than others. Taken together, this study provides a novel prediction system for synonymous mutations, called Syntool, which could be helpful in identifying candidate synonymous disease causing mutations.

## 1. Introduction

Next-generation sequencing technology has generated a vast amount of genomic data, but for clinical applications, interpretability of the results remains a major challenge. A central challenge in interpreting personal genomes data is how to identify pathogenic mutations by using their prior information or other phenotype information.

There is general lack of awareness about synonymous mutations, which is sometimes called “silent” mutations, due to no effect on amino acid change. Therefore, a corollary of this perception was that synonymous mutations would have no effect on the fitness of an organism and would be “neutral” during evolution [1]. Recent increase in our knowledge suggests that synonymous mutations could cause changes in protein expression, conformation, and function [2, 3]. For example, a number of studies show that synonymous mutations result in aberrant mRNA splicing, which can lead to human disease [4–6]. What is more, the lack of awareness about deleterious synonymous mutations can lead to misclassification of pathogenic variants. For example, nonsynonymous one was found in individuals with Treacher Collins syndrome and reported to be disease causing [7]. However, more recent pedigree analysis showed that the nonsynonymous mutation was not pathogenic, and one synonymous mutation was pathogenic [6].

The methods of deleterious prediction could be helpful in identifying candidate disease causing mutations. Although a number of such methods in terms of amino acid characteristics [8–12] can be used to identify pathogenic variation, synonymous variation does not affect amino acid sequence of a protein. Other presently several tools exist to predict mutations pathogenicity such as SIFT [13], PolyPhen [14], M-CAP [15], and REVEL [16]. T can predict nonsynonymous mutations or missense mutations pathogenicity but can not predict synonymous mutations pathogenicity.

Recently, a new set of tools that prioritize candidate disease causing genes without a priori disease knowledge has emerged. For example, RVIS [17] is a framework that ranks protein-coding genes based on their intolerance to functional variation by using sequence data from 6503 whole exome sequences from the NHLBI GO Exome Sequencing Project (ESP) [18]; RVIS is constructed by comparing the overall number of observed variants in a gene to the observed common functional variants. However, different functional variations lead to different degrees of deleterious effects. For synonymous and missense variation, Lek et al. [18] created a signed *Z* score for the deviation of observed counts from the expected number. Both RVIS and *Z* score compute different deviation amounts of observation from expectations such as chi-squared value or residuals value.

Both synonymous and missense variation are one-base substitution; however, synonymous variation does not cause any change in amino acid sequence while missense does. In other words, missense variation could be more deleterious than synonymous one. However, one previous study [19] concluded that nonsynonymous and synonymous coding SNPs show similar likelihood and effect size of human disease association. And synonymous mutation can be implicated in disease because of the effect on splicing and/or mRNA stability due to codon change as well as missense mutation. It means that missense mutation also can affect splicing and/or mRNA stability due to codon change [20, 21]. So synonymous score methods like ExAC synonymous *Z* score may be not complete to predict mutations pathogenicity.

Here we evaluate the intolerance of genic transcripts to single nucleotide substitution variation based on gnomAD 123136 individuals. By regressing all variations on common variations, we took the residual as the raw intolerance score. And then all ensemble transcript-based definition regions would be spited into several nonoverlapped subregions annotated by raw score; the minimum score of one subregion was defined as the subregion's intolerance score. The result shows that Syntool score we constructed clearly outperforms other tools in identifying HGMD Professional [22] “MD” tag synonymous mutations and ClinVar pathogenic mutations.

## 2. Methods

Ensemble's transcript definitions were adopted as our coding-sequence source data and each gene's expressed region was defined as analysis boundary.

*GnomAD Dataset*. The low quality SNP (single nucleotide polymorphisms) terms were filtered and “PASS” filter status of the gnomAD dataset was retained, and all SNPs were further annotated by Annovar [23]. Only those transcript-definition regions that sequenced at least a 10-fold depth mean coverage in 70% of their CDS in the gnomAD dataset were ranked. The gnomAD variant dataset was annotated by Annovar [23] with Sequence Ontology (SO) [24] variant function definitions.

Of these variant annotations, the variant hit exon variant (SO:0001791), splicing variant (SO:0001568), noncoding transcript variant (SO:0001619), 5′ prime UTR variant (SO:0001623), 3′ prime UTR variant (SO:0001624), intron variant (SO:0001627), upstream gene variant (SO:0001631), downstream gene variant (SO:0001632), and intergenic variant (SO:0001628), only exon variant (SO:0001791) was considered.

The coding variant annotations include frameshift elongation (SO:0001909), frameshift truncation (SO:0001910), frameshift variant (SO:0001589), stop gained (SO:0001587), stop lost (SO:0001578), inframe insertion (SO:0001821), inframe deletion (SO:0001822), inframe variant (SO:0001650), missense variant (SO:0001583), synonymous variant (SO:0001819), and sequence variant (SO:0001060). Missense and synonymous ones were considered and defined as single nucleotide substitution variation.

*Minor Allele Frequency*. We defined cutoff value as 0.1%. If the variation's MAF value >cutoff, we defined the variation as common variation.

*Intolerance Score*. We let *X* as the sum of single nucleotide substitution variations (regardless of frequency in the population) observed in the transcript-definition region and *Y* as the total number of common single nucleotide substitution variations (the MAF value >0.1%). By fitting the regression model of *Y* on *X*, we took the residual as the raw intolerance score. And then all ensemble transcript-definition regions would be spited into several nonoverlapped subregions annotated by raw score; the minimum score of one subregion was defined as the subregion's intolerance score and those regions which shared the same raw score would be merged. Finally, a total of 24799 nonoverlap region-based intolerance scores by their intolerance to single nucleotide substitution variation for synonymous mutations predictions (Syntool) was constructed.

## 3. Results

### 3.1. A Total of 24799 Nonoverlap Region-Based Intolerance Scores by Their Intolerance to Single Nucleotide Substitution Variation for Synonymous Mutations Predictions (Syntool)

After fitting the regression model of sum single nucleotide substitution variations number on common single nucleotide substitution variations (whose MAF value >0.1%) number, we derived the intolerance score to single nucleotide substitution variation for synonymous mutations predictions (Syntool score).

The size of the raw score depends on the transcript definitions we adopted which contains 51679 transcripts. And then all ensemble transcript-definition regions would be spited into several nonoverlapped subregions annotated by raw score; the minimum score of one subregion was defined as the subregion's intolerance score and those regions which shared the same raw score would be merged. Finally, our database covered 0.9 G bases (31% of 2.9 G human reference) with 24799 regions. Thus, Syntool score (*S*) was divided by an estimate of its standard deviation and accounts for differences in variability that come with differing mutational burdens. If *S* = 0, the region of genomic has the average number of single nucleotide substitution variants given its total common mutational; if *S* > 0, the region of genomic has more single nucleotide substitution variants than predicted; if *S* < 0, it has less single nucleotide substitution variations than predicted. The differences between observed value and fitted value could reflect the differences of purifying selection. That means if *S* < 0, the variation of the gene suffers purifying selection to become rare variation, and the mutation that occurs in these regions could be predicted more likely to cause certain kinds of diseases.

Here, we have adopted *p * = * *0.1% MAF as threshold for defining common variants in the gnomAD database. However, we also explored the behavior of the raw score for *p* of 0.01% and 1% and found both of these to be strongly correlated with *p * = * *0.1% (Pearson's *r*  =  0.997 and Pearson's *r*  =  0.996, resp.).

### 3.2. Intolerance to Single Nucleotide Substitution Variation for Synonymous Mutations Predictions (Syntool) Score Can Discriminate Disease Causing Mutation Much Better

**Figure 1:**
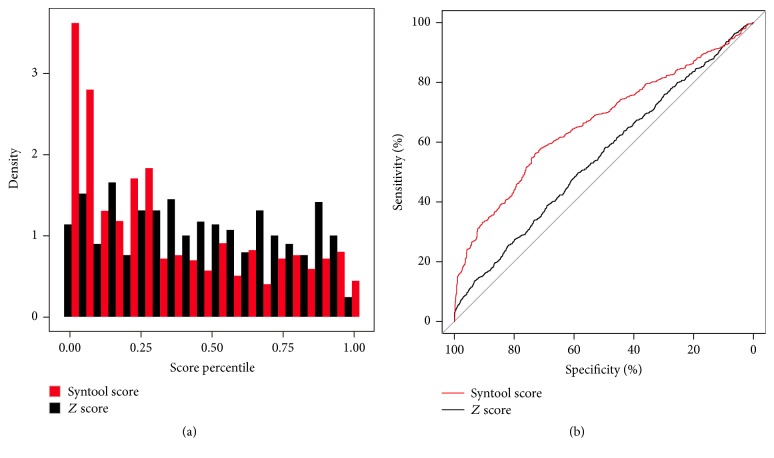
Comparison between Syntool score and ExAC gene constraint synonymous *Z* score. (a) Distribution of the HGMD disease causing synonymous mutation and ClinVar pathogenic mutations in the intolerant score percentile. The lower the variation score percentiles, the more intolerant the regions/genes to variation. (b) ROC curves of Syntool score and ExAC gene constraint synonymous *Z* score to discriminate the genomics region/gene whether there is synonymous disease causing mutation. The lower the variation score percentiles, the more intolerant the regions/genes to variation.

To assess whether the Syntool score can discriminate variations associated with diseases much better, we analyze the correlation between Syntool score and mutation which is related with disease (Figure 1(a)). We annotated the HGMD Professional database “DM” tag mutations and ClinVar pathogenic mutations; we got disease synonymous mutation list (*n* = 1218) and gene list (*n* = 647). Among those 1037 mutations could be annotated by the Syntool score, with 68.5% true positive rate while 632 genes could be annotated by ExAC synonymous *Z* score, with 53.0% true positive rate. The Syntool score seems to perform much better. For example, previous studies [5, 6] found two synonymous mutations in TCOF1 and MPZ gene causing Charcot-Marie-Tooth disease type 1b and Treacher Collins syndrome.

Syntool score is a novel region-based intolerance score for synonymous mutations predictions; thus the power about discriminating the genomics region whether there is synonymous disease causing mutation is necessary. However, the small number of disease causing synonymous mutations results in the lower AUC (Figure 1(b), AUC = 66.45% of Syntool score while ExAC synonymous *Z* score AUC = 55.33%).

## 4. Discussion

Prioritizing candidate disease causing genes remains a major challenge. For clinical applications, interpretability of the variation especially synonymous variation remains a major challenge.

There is general lack of awareness about synonymous mutations, which is sometimes called “silent” mutations, due to no effect on amino acid change. However the recent increasing knowledge suggests that synonymous mutations could cause changes in protein expression, conformation, and function [2]. What is more, the lack of awareness about deleterious synonymous mutations can lead to misclassification of pathogenic variants. The Syntool score could be helpful in identifying candidate synonymous disease causing mutations. Comparing with ExAC gene constraint synonymous *Z* score, the Syntool score we derived can better discriminate synonymous variation in Human Gene Mutation Database (HGMD Professional) and ClinVar database whether there is pathogenic mutation. And the Syntool performed more sensitively in the prediction of synonymous mutation than gene level of ExAC gene constraint synonymous *Z* score.

## Additional Points

*Availability and Implementation*. Check https://github.com/zhangtongda/Syntool.
